# Sevoflurane impedes neuropathic pain by maintaining endoplasmic reticulum stress and oxidative stress homeostasis through inhibiting the activation of the PLCγ/CaMKII/IP3R signaling pathway

**DOI:** 10.18632/aging.206001

**Published:** 2024-07-05

**Authors:** An Xie, Xianjie Zhang, Feng Ju, Yukai Zhou, Dan Wu, Jia Han

**Affiliations:** 1 Department of Anesthesiology, People’s Hospital of Deyang, Deyang, Sichuan, China

**Keywords:** chronic constriction injury of sciatic nerve, neuropathic pain, sevoflurane, calcium influx, oxidative stress

## Abstract

Objective: To investigate the effect of sevoflurane on neuropathic pain induced by chronic constriction injury (CCI) of sciatic nerve in mice, and to elucidate its mechanism by animal experiments.

Methods and Results: Thirty-two C57BL/6 mice were randomly divided into four groups: Sham group, Model group, Control group and Sevoflurane group. First, a mouse model of neuropathic pain was established. Then, the mice in each group were killed on Day 14 after operation to harvest the enlarged lumbosacral spinal cord. In contrast with the Model group, the Sevoflurane group displayed a significantly increased paw withdrawal mechanical threshold (PWMT) and significantly prolonged paw withdrawal thermal latency (PWTL) from Day 5 after operation. The morphological changes of lumbosacral spinal cord were observed by hematoxylin-eosin (HE) staining and transmission electron microscopy. Pathological results showed that sevoflurane reduced nuclear pyknosis in lumbosacral spinal cord tissue, with a large number of mitochondrial crista disappearance and mitochondrial swelling. The results of Western blotting showed that sevoflurane significantly decreased the protein expressions of phosphorylated phospholipase Cγ (p-PLCγ), phosphorylated calcium/calmodulin-dependent protein kinase II (p-CaMKII) and phosphorylated inositol 1,4,5-triphosphate receptor (p-IP3R), and reduced the protein expressions of endoplasmic reticulum (ER) stress proteins glucose-regulated protein 78 (GRP78) and GRP94, oxidative stress-related proteins P22 and P47 and inflammatory factors nucleotide-binding oligomerization domain-like receptor protein 3 (NLRP3), interleukin-1 β (IL-1β), and tumor necrosis factor-α (TNF-α).

Conclusions: Sevoflurane inhibits neuropathic pain by maintaining ER stress and oxidative stress homeostasis through inhibiting the activation of the PLCγ/CaMKII/IP3R signaling pathway.

## INTRODUCTION

Neuropathic pain, a common kind of chronic pain in clinic, is caused by the disease or injury of somatosensory system, which has serious adverse effects on the spirit and the quality of sleep and life of patients. Neuropathic pain is persistent and refractory, and its recurrence places a heavy burden on the family and society [1]. At the moment, analgesics, antidepressants, antiepileptic medications, etc., are some of the most frequently used medications for neuropathic pain [2]. However, these drugs do not provide optimal therapeutic effects and lead to many side effects [3, 4]. Therefore, it is necessary to explore new treatment ideas.

The mechanism behind neuropathic pain is intricate. One of the key mechanisms of neuropathic pain is neurogenic inflammation following nerve injury. Previous studies have shown that after spinal nerve injury, microglia and astrocytes activate and release such inflammatory factors as interleukin-6 (IL-6), IL-1β and tumor necrosis factor-α (TNF-α), thus promoting the occurrence of neuropathic pain [5, 6]. G-protein coupled receptors (GPCRs) are a class of transmembrane receptors widely involved in intracellular signal transduction and regulation. Many neuromodulators and neurotransmitters regulate cell generation and signaling through GPCR-mediated calcium influx. Excess calcium influx, on the other hand, can result in calcium imbalance, which affects mitochondrial function and induces such pathological states as oxidative stress and neuropathic pain [7]. GPCRs serve as a heterotrimer that releases Gβγ and Gα upon activation [8]. It has been shown that the release of Gβγ eventually leads to the activation of phospholipase Cγ (PLCγ) [9, 10]. The activation of PLCγ produces a biophysical composition of three distinct inositol 1,4,5-triphosphate receptors (IP3Rs) that trigger the release of successive calcium ions from the endoplasmic reticulum (ER) through the mitochondrial-associated ER membrane (MAM) to the mitochondria.

Sevoflurane is a drug widely used for both general anesthesia and the management of neuropathic pain. Sevoflurane is characterized by highly efficient, rapid and safe potency, and its effect in the treatment of neuropathic pain has been widely recognized [11]. Early studies argued that sevoflurane inhibits pain signaling by directly acting on neurons or intra-neural glial cells [12]. However, it was discovered that the mechanism of sevoflurane in the treatment of neuropathic pain is more complicated.

Therefore, it was believed that the role of sevoflurane is highly overlapping with the pathogenesis of neuropathic pain, and it was speculated that sevoflurane should have a therapeutic effect on neuropathic pain with no obvious side effects. In this study, animal models of neuropathic pain were established, and associated experiments were conducted to lay the groundwork for future research into the mechanisms underlying neuropathic pain, and to create novel applications and concepts for sevoflurane.

## RESULTS

### Comparison of PWMT among four groups

**Figure 1:**
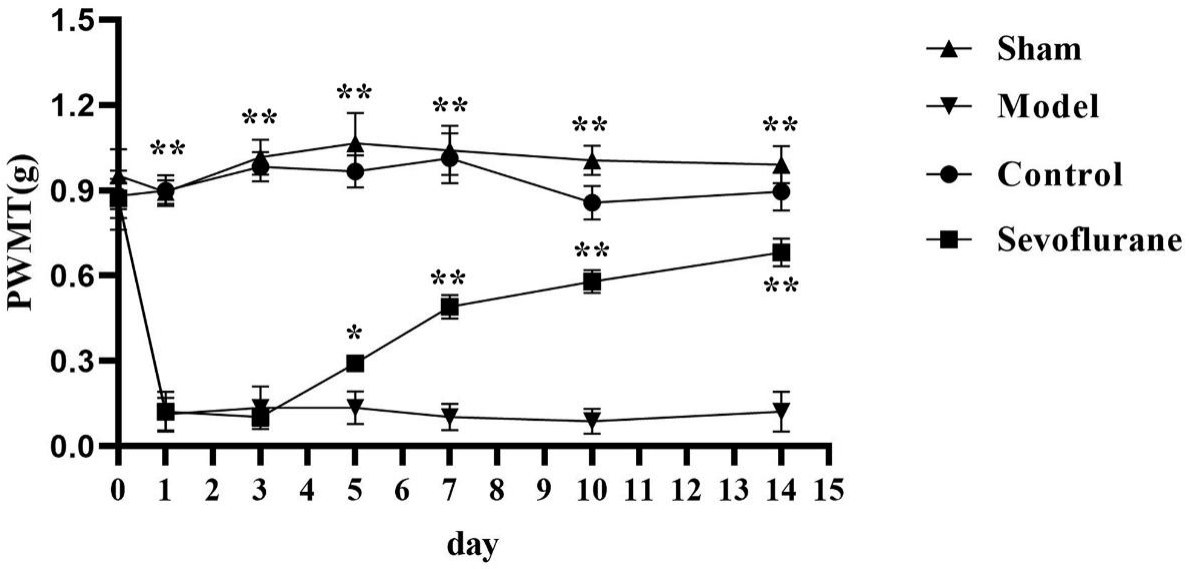
**Comparison of PWMT among four groups of mice.** Sham group vs. Model group, Model group vs. Sevoflurane group, Control group vs. Sevoflurane group, **P<0.01, *P<0.05, N=8.

The PWMT of mice in each group was tested before operation and on Days 1, 3, 5, 7, 10 and 14 after operation. From Day 1 after operation, the PWMT of mice was significantly lower in the Model group than that in the Sham group (P<0.05). Compared with that in the Model group, the PWMT was significantly increased in the Sevoflurane group from Day 5 after operation (P<0.05). The PWMT was significantly reduced in the Sevoflurane group compared with that in the Control group (Figure 1).

### Comparison of PWTL among four groups

**Figure 2:**
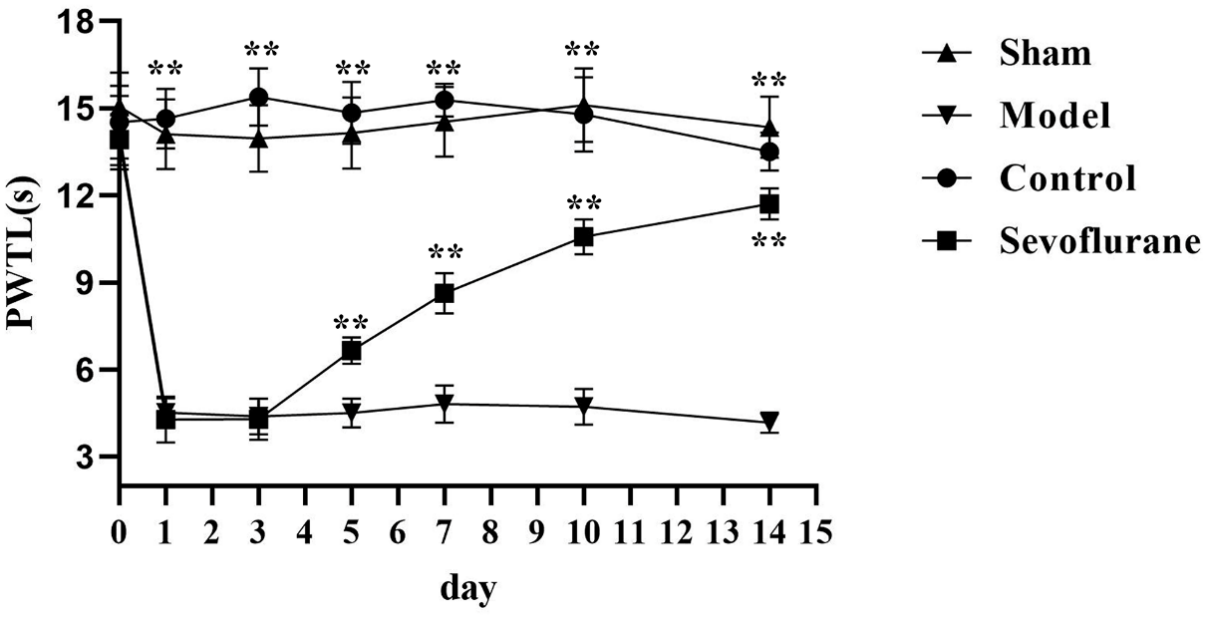
**Comparison of PWTL among four groups of mice.** Sham group vs. Model group, Model group vs. Sevoflurane group, Control group vs. Sevoflurane group, **P<0.01, N=8.

The PWTL was measured before operation and on Days 1, 3, 5, 7, 10 and 14 after operation. The results showed that the PWTL was significantly shortened in the Model group from Day 1 after operation compared with that in the Sham group, with a statistically significant difference (P<0.05). The PWTL in the Sevoflurane group was significantly prolonged compared with that in the Model group from Day 5 after operation (P<0.05), but significantly shortened compared with that in the Control group (Figure 2).

### Pathological changes in mice

**Figure 3:**
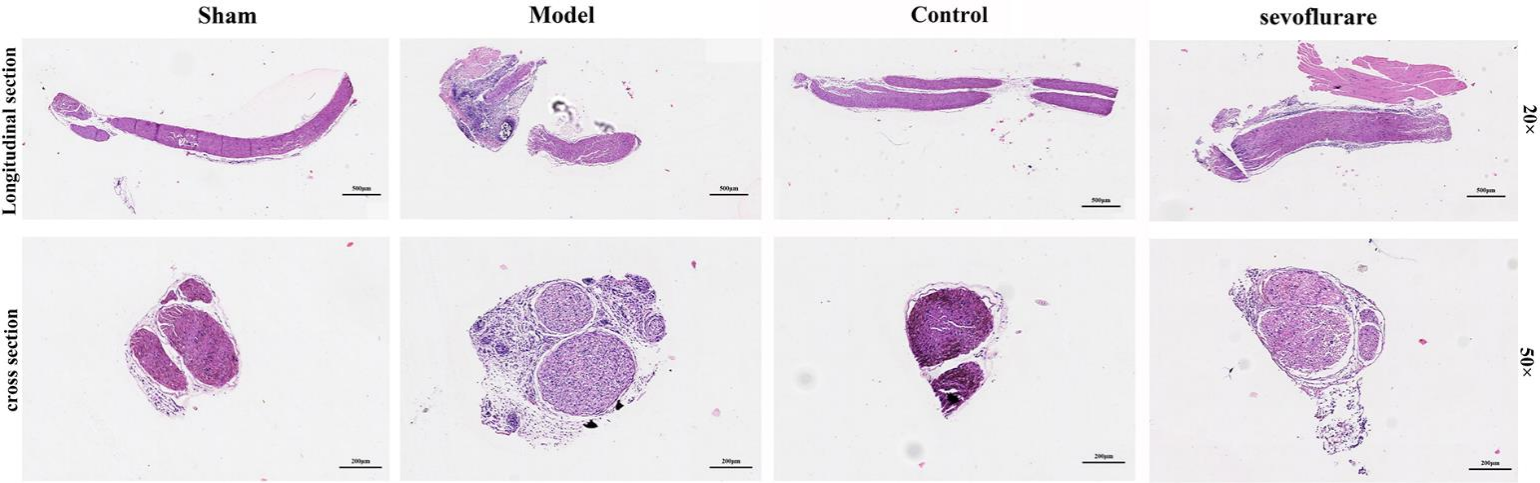
Sevoflurane could relieve the damage caused by CCI of the sciatic nerve, N=8.

HE staining was conducted to detect pathological changes of sciatic nerve tissues. The results showed that in the Sham group and Control group, there were normal morphology, clear boundary, round nucleus and obvious nucleolus in terms of arrangement of cells. In the Model group, the cells were arranged disorderly, atrophic and degenerated, the nuclear boundary was not clear, the nucleolus disappeared, and interstitial edema was observed. Compared with those in the Model group, the cells in the Sevoflurane group were arranged more orderly, with a lower degree of atrophy, a clearer nuclear boundary, and a more prominent nucleus (Figure 3). The results suggested that sevoflurane can alleviate the injury caused by chronic constriction injury (CCI) of sciatic nerve.

### Observation of ultrastructural changes in mouse tissues by transmission electron microscopy

**Figure 4:**
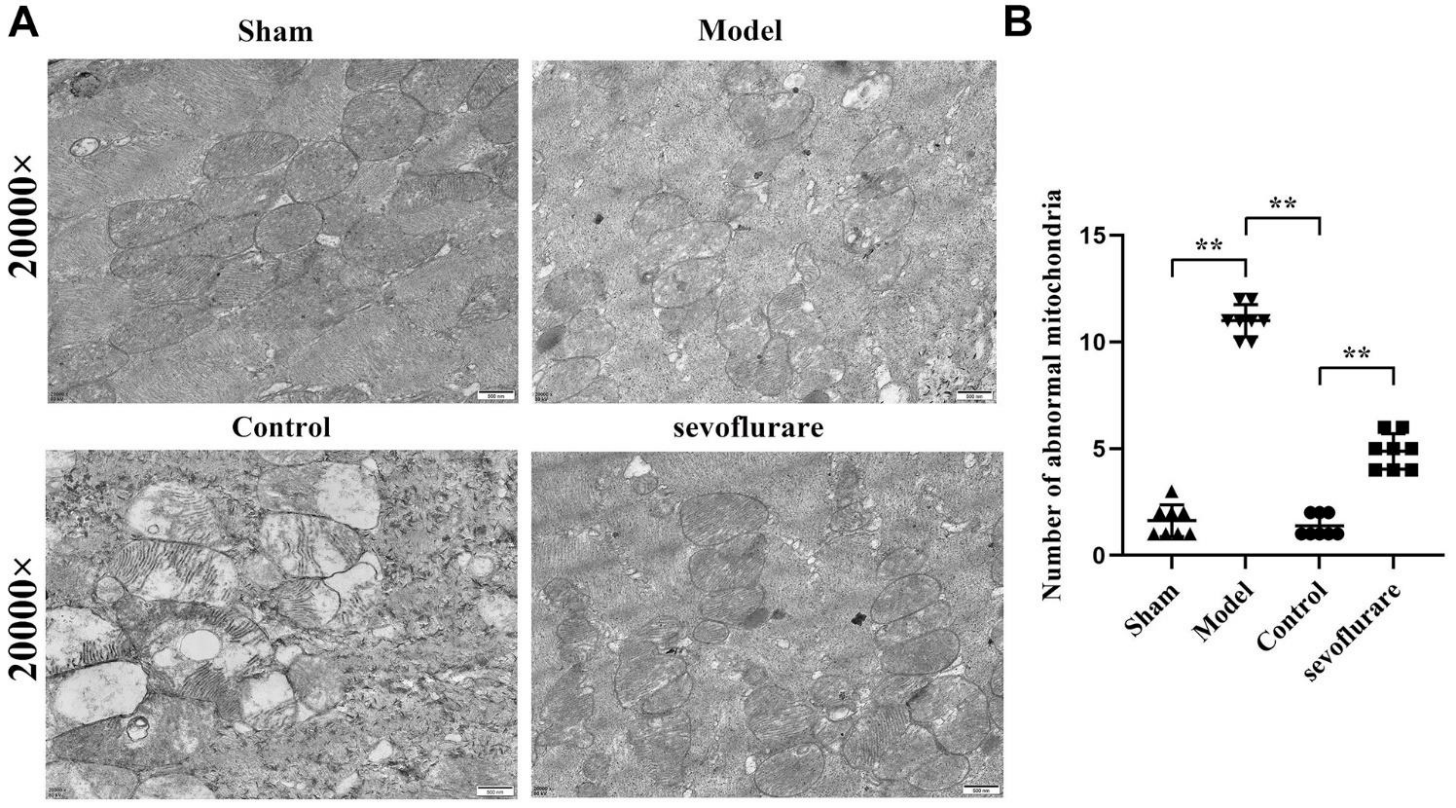
**Representative transmission electron microscopic images of mouse lumbosacral spinal cord tissue.** (**A**) Results of transmission electron microscopy, (**B**) mitochondrial number statistics. Sham group vs. Model group, Model group vs. Sevoflurane group, Control group vs. Sevoflurane group, N=8.

With a transmission electron microscope, the sub organelle in the lumbosacral spinal cord tissue of mice can be observed to the maximum extent. The results manifested that in the Sham group and Control group, mitochondria were oval or rod-shaped, surrounded by a double membrane, and the inner membrane protruded inward into a flat ridge. The spine was perpendicular to the long axis of mitochondria and had a large number of mitochondria. Compared with the Sham group, the Model group had many abnormal mitochondria, including vacuolization, swelling, decreased matrix density, and mitochondrial ridge rupture. Some severely damaged mitochondria and inner and outer membrane defects of cristae had completely disappeared. The number of abnormal mitochondria in the Sevoflurane group was significantly reduced compared to that in the Model group, and the situation was better than that in the Model group. Compared with that in the Control group, the number of mitochondria in the Sevoflurane group was significantly reduced (Figure 4), with statistical differences (P<0.01). It suggested that sevoflurane can alleviate the cellular damage caused by CCI of the sciatic nerve.

### Sevoflurane reduced calcium influx and ER stress in CCI of sciatic nerve

**Figure 5:**
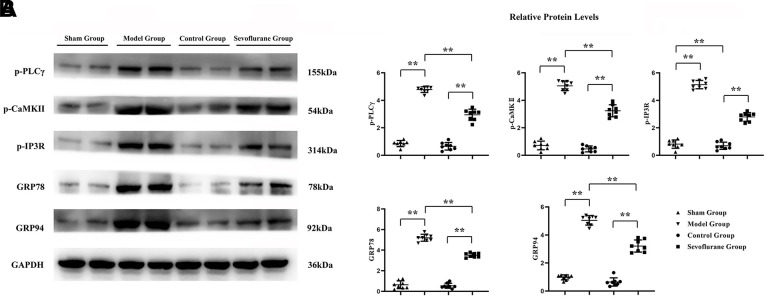
**Sevoflurane could relieve calcium influx and ER stress.** (**A**) Protein bands of p-PLCγ, p-CaMKII, p-IP3R, GRP78 and GRP94 in Sham group, Model group and Sevoflurane group, (**B**) relative protein expressions of p-PLCγ, p-CaMKII, p-IP3R, GRP78 and GRP94 in Sham group, Model group and Sevoflurane group. Sham group vs. Model group, Model group vs. Sevoflurane group, Control group vs. Sevoflurane group, **P<0.01, N=8.

Western blotting was carried out to detect the expression changes of GPCR-mediated proteins (p-PLCγ, p-CaMKII and p-IP3R) and ER stress proteins (GRP78 and GRP94) in sciatic nerve tissue. The results showed that the expressions of p-PLCγ, p-CaMKII, p-IP3R, GRP78, and GRP94 in the Model group increased significantly compared with those in the Sham group. The relative protein expression levels of p-PLCγ, p-CaMKII, p-IP3R, GRP78 and GRP94 in the Sevoflurane group were significantly reduced compared with those in the Model group, but they were significantly increased compared with those in the Control group (Figure 5). The data had statistical differences (P<0.01). The results indicated that sevoflurane represses neuropathic pain by maintaining ER stress and oxidative stress homeostasis through inhibiting the activation of the PLCγ/CaMKII/IP3R signaling pathway.

### Sevoflurane reduced oxidative stress and inflammatory responses in sciatic nerve tissue

**Figure 6:**
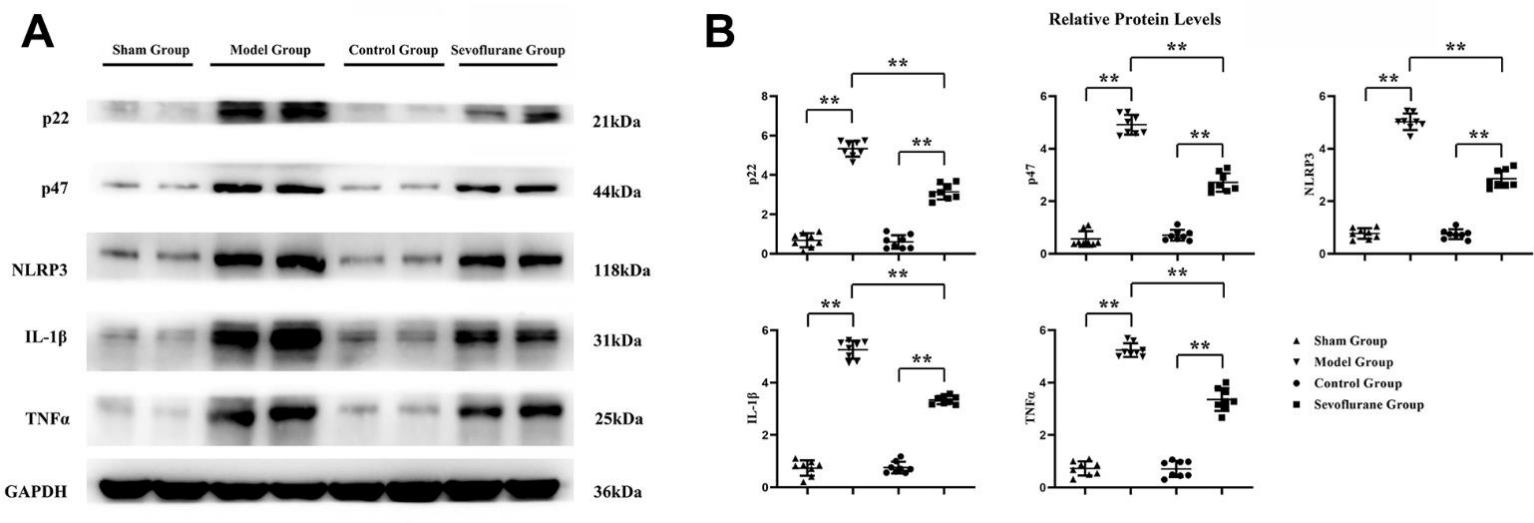
**Sevoflurane could reduce oxidative stress and inflammation.** (**A**) Protein bands of P22, P47, NLRP3, IL-1β and TNF-α in Sham group, Model group and Sevoflurane group, (**B**) relative protein expressions of P22, P47, NLRP3, IL-1β and TNF-α in Sham group, Model group and Sevoflurane group. Sham group vs. Model group, Model group vs. Sevoflurane group, Control group vs. Sevoflurane group, **P<0.01, N=8.

The expressions of oxidative stress proteins and inflammatory factors in sciatic nerve tissue were measured by Western blotting. The results revealed that the expressions of P22, P47, NLRP3, IL-1β and TNF-α were significantly higher in the Model group than those in the Sham group. Compared with those in the Model group, the expressions of P22, P47, NLRP3, IL-1β and TNF-α in the Sevoflurane group were significantly reduced, while such expressions significantly rose compared with those in the Control group (Figure 6). The data had statistical differences (P<0.01). It suggested that sevoflurane may reduce oxidative stress and inflammation in CCI of sciatic nerve tissue.

### *In vitro* experiments verified that sevoflurane inhibits neuropathic pain by repressing the activation of the PLCγ/CaMKII/IP3R signaling pathway to maintain ER stress and oxidative stress homeostasis

**Figure 7:**
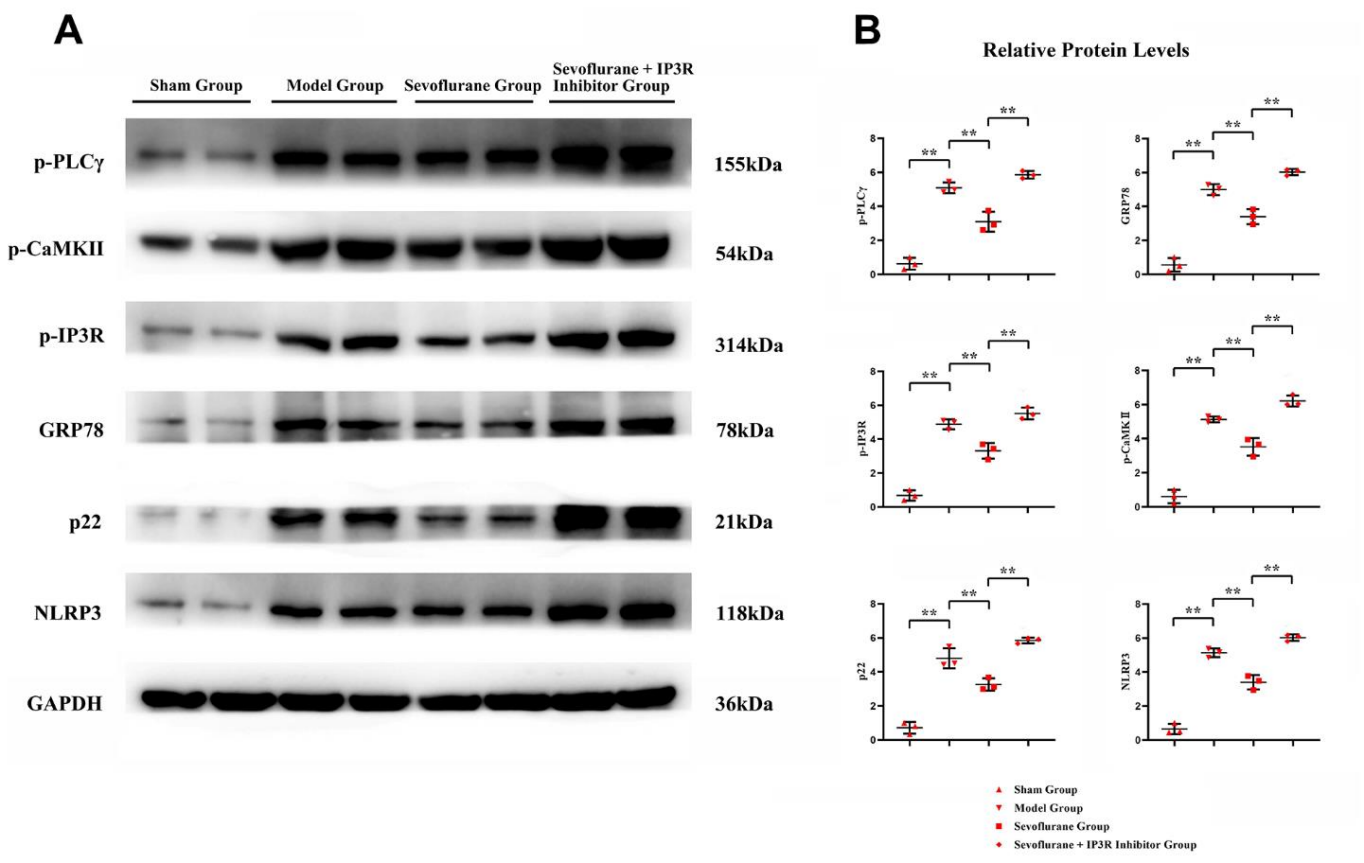
***In vitro* experiments verified that sevoflurane could mediate the PLCγ/CaMKII/IP3R signaling pathway to inhibit ER stress, oxidative stress and inflammatory response.** (**A**) Protein bands of p-PLCγ, p-CaMKII, p-IP3R, GRP78, P22 and NLRP3 in each group, (**B**) relative protein expressions of p-PLCγ, p-CaMKII, p-IP3R, GRP78, P22 and NLRP3 in each group. Sham group vs. Model group, Model group vs. Sevoflurane group, Sevoflurane group vs. Sevoflurane + IP3R Inhibitor group, **P<0.01, N=3.

**Figure 8:**
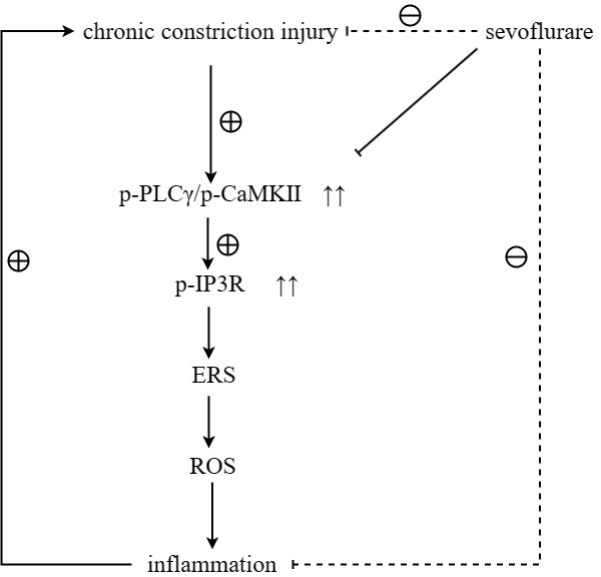
Sevoflurane maintained ER stress and oxidative stress homeostasis by inhibiting the activation of the PLCγ/CaMKII/IP3R signaling pathway, thereby repressing neuropathic pain.

To verify the conclusions of *in vivo* experiments, *in vitro* cell experiments were performed and it was found that the relative protein expressions of p-PLCγ, p-CaMKII, p-IP3R, GRP78, P22 and NLRP3 were significantly higher in the Model group than those in the Control group, and in the Model group and Sevoflurane group than those in the Model group. The relative protein expression of NLRP3 was significantly reduced, and the expression of the above proteins was significantly increased after the addition of the IP3R agonist Adenophostin A hexasodium salt (Figure 7). This result verified that sevoflurane maintains ER stress and oxidative stress homeostasis by inhibiting the activation of the PLCγ/CaMKII/IP3R signaling pathway (Figure 8).

## DISCUSSION

CCI of the sciatic nerve occurs when the sciatic nerve is compressed or constricted, causing long-lasting nerve damage. Disc herniation, lumbar spinal canal stenosis, and bone spurs are all common reasons that can cause surrounding soft tissue and nerve compression, resulting in pain and other symptoms [13]. Therefore, it is necessary to explore the specific mechanism of CCI of the sciatic nerve.

Sevoflurane is a colorless and transparent liquid with an anesthetic effect, which is one of the most widely used general anesthetic in the medical field. It can be applied by steam inhalation or injection [14]. Due to its quick onset, controllable depth of anesthesia, and minimal impact on the cardiovascular and respiratory systems, sevoflurane is frequently used in surgical and emergency anesthesia. As a commonly used general anesthetic, sevoflurane is usually administered by the inhalation route due to its faster absorption, *i.e.*, its gas molecules can be quickly absorbed into the bloodstream through the respiratory tract. In addition, sevoflurane has a large volume of distribution, *i.e.*, it is widely distributed in the body, including brain tissue, muscle, adipose tissue, and other tissues. Sevoflurane is mainly oxidized and metabolized in the liver to produce water-soluble metabolites, which are then excreted in urine. During anesthesia, sevoflurane is gradually metabolized and degraded by the liver. After the metabolism of sevoflurane, the metabolites are mainly excreted through urine, and a small amount is also excreted through exhaled gas. Therefore, in this study, it was found by HE staining and electron microscopy that low-dose sevoflurane could attenuate the tissue damage caused by CCI of sciatic nerve.

CCI of the sciatic nerve can induce calcium influx, which in turn causes oxidative stress and inflammation. GPCRs are heterotrimers that release Gβγ and Gα upon activation [15, 16]. The release of Gβγ ultimately gives rise to the activation of PLCγ [9], after which the biophysical composition of three distinct IP3Rs is generated, triggering the release of continuous calcium 2+ from the ER to mitochondria via the MAM [17, 18], thus causing oxidative stress and inflammation. Sevoflurane has been shown to be involved in the activation of phosphatidylinositol 3 kinase (PI3K). Therefore, it was hypothesized that sevoflurane could inhibit the GPCR-induced signaling pathway, and research was carried to study the mechanism of pain induced by CCI of sciatic nerve to develop new applications and provide new inspirations for sevoflurane. Western blotting results showed that the expressions of p-PLCγ, p-CaMKII, p-IP3R, GRP78, GRP94, P22, P47, NLRP3, IL-1β and TNF-α in the Model group were significantly higher than those in the Sham group. Compared with those in the Model group, the expressions of p-PLCγ, p-CaMKII, p-IP3R, GRP78, GRP94, P22, P47, NLRP3 and TNF-α significantly declined in the Sevoflurane group. Compared with those in the Control group, the relative protein expression levels of p-PLCγ, p-CaMKII, p-IP3R, GRP78, GRP94, P22, P47, NLRP3 and TNF-α significantly rose in the Sevoflurane group, indicating that sevoflurane maintains ER stress and oxidative stress homeostasis by inhibiting the activation of the PLCγ/CaMKII/IP3R signaling pathway. Besides, this finding was verified in this study through *in vitro* experiments. Based on this, sevoflurane may be effective in modulating peripheral and central immune responses to neuropathic pain.

In this study, a neuropathic pain model was established in mice and the effect of sevoflurane on neuropathic pain in mice was observed. The results showed that sevoflurane impedes neuropathic pain by maintaining ER stress and oxidative stress homeostasis through inhibiting the activation of the PLCγ/CaMKII/IP3R signaling pathway. The application of sevoflurane in the treatment of neuropathic pain still needs to be further verified by more basic and clinical trials.

## MATERIALS AND METHODS

### Establishment and grouping of neuropathic pain model in mice

Thirty-two male C57BL/6 mice weighing 20-25 g were randomly divided into four groups: Sham group (mice underwent surgery without ligation and were then given 0.2 mL/d of normal saline by nasal feeding), Model group (mice were given normal saline at 0.2 mL/d through nasal feeding after modeling), Control group (mice did not receive any treatment and were then given 0.2 mL/d of normal saline by nasogastric feeding), and Sevoflurane group (mice were given 40 mg/(kg·d) of sevoflurane through nasal feeding after modeling). All the mice were fed normally before operation. The anesthesia process lasted for 30 min and the mouse state was observed at all times, and then the experiment was started the next day. Process of modeling: after the mice were anesthetized with isoflurane, the skin was cut at the high position of the right femoral segment of the mice, the muscles were bluntly separated, and the sciatic nerve trunk was exposed. Then, the sciatic nerve trunk was ligated with 7-0 nylon silk thread for 3 times at an interval of 1 mm. After ligation, the sciatic nerve was anatomically returned and sutured to the skin layer by layer. After anesthesia in the Sham group, only the skin was cut open to expose the left sciatic nerve without clamping, and the corresponding site was marked in the same way and sutured. The whole process was performed under aseptic operation. The mice were raised in single cage after operation. If the mice have lower limb paralysis, dyskinesia or death after operation, they need to be removed and replaced with new mice for modeling. The criteria for successful modeling were as follows: the mice had spontaneous pain, triggered pain, hyperalgesia, hypoesthesia and paraesthesia. The mice in the four groups were administered by nasal feeding from Day 3 to Day 14 after operation.

### Cell culture

The RSC96 cell line was purchased from Procell (Wuhan, China) was cultured in DMEM and added with 10% FPS and 1% bispecific antibody solution. Cells were divided into Control group, Model group, Sevoflurane group, and Sevoflurane + IP3R inhibitor (Adenophostin A hexasodium Salt, 10 nM) group. The cells in the Model group, Sevoflurane group and Sevoflurane+IP3R inhibitor group were added with LPS (10 μg/mL), and then the culture plates were placed in a closed container with gas mixture. The inlet of the closed container was connected to the outlet of an anesthesia gas detector (Ohmeda 7100) at 2 L/mm, and the volatile output concentration and the heptaoxygen concentration were set to 3% and 2.5%, respectively. After stopping the gas mixture blowing in, the inlet and outlet of the closed container were immediately clamped, and the closed container was placed into a constant temperature incubator at 37° C, followed by incubation for 1 h. Thereafter, the cells were put into a 5% CO_2_ constant temperature incubator at 37° C, and eluted for 10 min.

### Determination of the mechanical withdrawal reflex in mice

The paw withdrawal mechanical threshold (PWMT) in mice was measured before operation and on Days 1, 3, 5, 7, 10 and 14 after operation by Von-Frey test. In brief, the mice were put into a hollow metal cage for 20-30 min to fully adapt to the environment, and then the manual Von-Frey was used to stimulate the middle metatarsus of the mice after modeling. Next, the up and down method, i.e., the way of decreasing or increasing the force filament, was adopted, and repeated 10 times with each time for 6-8 s. The interval between every two tests shall be more than 15 s. To prevent damage to mice, the experimental interval was 10 min. Five or more licking or withdrawal reactions in 10 tests indicated the positive result, and the PWMT was the minimum force of positive result.

### Determination of thermal withdrawal reflex latency in mice

The paw withdrawal thermal latency (PWTL) of mice was evaluated by the Hargreaves test before operation and on Days 1, 3, 5, 7, 10 and 14 after operation. Briefly, the hind plantar of mice was stimulated with radiation emitted by halogen tungsten lamp. The thermal pain threshold of mice was the time of licking the paw, withdrawing the paw or jumping away. During the test, mice were placed in an elevated translucent plexiglass cage with the specification of 20 cm×20 cm×25 cm, and adapted to the environment for about 30 min. The room temperature was controlled at (24±2) °C, and the environment was kept quiet. The mice were irradiated with radiant heat from a 50 W, 8 V bulb. To prevent damage to the plantar of mice, a cut-off time of 30 s and an interval of 10 min between ipsilateral plantar irradiation were set. Each mouse was tested 6 times.

### Hematoxylin-eosin (HE) staining

The prepared paraffin sections were completely immersed in the deparaffinizing agent, dehydrated, soaked in hematoxylin solution for 5 min, and washed with running water, followed by differentiation with 1% hydrochloric acid alcohol for tens of seconds and then observation under the microscope after the disappearance of blue cytoplasm to terminate the differentiation. Thereafter, the sections were added with 1% ammonia water to turn them to blue for tens of seconds until the nuclei became blue, followed by washing with running water. The sections were dipped in eosin solution for 30 s, and rinsed with running water. Afterwards, the sections were dehydrated, transparentized and sealed.

### Transmission electron microscopy

The samples were fixed overnight in a 0.1 M sodium bicarbonate buffer of 2.5% glutaraldehyde, and then in a 0.1 M sodium coconut buffer with 1% osmium tetroxide. Next, they were dehydrated, and embedded in EPON-812 resin in epoxy propane. Thereafter, semi thin cross-sections were stained with 0.5% toluidine blue O, and bright-field microscopic images were captured using Zeiss Axioskop equipped with an RT slider point camera. Ultrathin cross-sections were stained with uranyl acetate and lead citrate, and the images were captured by an electron microscope (JEM-1400).

### Western blotting

The total protein was extracted from sciatic nerve tissue with RIPA lysis buffer and its concentration was determined by BCA method. After separation through SDS-PAGE, the target protein was transferred to a PVDF membrane. Thereafter, the membrane was blocked for 2h at room temperature, and reacted with corresponding primary antibodies against phosphorylated (p)-PLCγ (Abcam, UK, ab81284, 1: 2000), phosphorylated calcium/calmodulin-dependent protein kinase II (p-CaMKII) (Abcam, ab171095, 1: 2000), p-IP3R (Cell Signaling, USA, 8548, 1: 1000), glucose-regulated protein 78 (GRP78) (Abcam, ab171089, 1: 1000), GRP94 (Abcam, ab210960, 1: 1000), P22 (Abcam, ab75941, 1: 1000), P47 (Abcam, ab308256, 1: 1000), nucleotide-binding oligomerization domain-like receptor protein 3 (NLRP3) (Abcam, ab263899, 1: 1000), IL-1β (Abcam, ab283818, 1: 1000), TNF-α (Abeam, ab205587, 1: 1000) and GAPDH (Abcam, ab2293, 1: 2500) at 4° C overnight. The next day, the excess primary antibody was washed off and the corresponding secondary antibodies were added for incubation for 2 h at room temperature. After removal of excess secondary antibody, chemiluminescent solution was added dropwise, images were acquired using a gel imaging system, and gray values were analyzed using BandScan.

### Statistical analysis

Data were processed using SPSS22.0 software. Measurement data were expressed as mean ± standard deviation. One-way analysis of variance was adopted for comparison between groups, and repeated measures analysis of variance was employed for behavioral comparison. P<0.05 indicated a statistically significant difference.

## References

[1] Finnerup NB, Kuner R, Jensen TS. Neuropathic Pain: From Mechanisms to Treatment. Physiol Rev. 2021; 101:259–301. 10.1152/physrev.00045.2019 32584191

[2] St John Smith E. Advances in understanding nociception and neuropathic pain. J Neurol. 2018; 265:231–8. 10.1007/s00415-017-8641-6 29032407 PMC5808094

[3] Szok D, Tajti J, Nyári A, Vécsei L. Therapeutic Approaches for Peripheral and Central Neuropathic Pain. Behav Neurol. 2019; 2019:8685954. 10.1155/2019/8685954 31871494 PMC6906810

[4] Cavalli E, Mammana S, Nicoletti F, Bramanti P, Mazzon E. The neuropathic pain: An overview of the current treatment and future therapeutic approaches. Int J Immunopathol Pharmacol. 2019; 33:2058738419838383. 10.1177/2058738419838383 30900486 PMC6431761

[5] Campbell JN, Meyer RA. Mechanisms of neuropathic pain. Neuron. 2006; 52:77–92. 10.1016/j.neuron.2006.09.021 17015228 PMC1810425

[6] Baron R, Binder A, Attal N, Casale R, Dickenson AH, Treede RD. Neuropathic low back pain in clinical practice. Eur J Pain. 2016; 20:861–73. 10.1002/ejp.838 26935254 PMC5069616

[7] Hauser AS, Attwood MM, Rask-Andersen M, Schiöth HB, Gloriam DE. Trends in GPCR drug discovery: new agents, targets and indications. Nat Rev Drug Discov. 2017; 16:829–42. 10.1038/nrd.2017.178 29075003 PMC6882681

[8] Wang J, Hanada K, Gareri C, Rockman HA. Mechanoactivation of the angiotensin II type 1 receptor induces β-arrestin-biased signaling through Gα_i_ coupling. J Cell Biochem. 2018; 119:3586–97. 10.1002/jcb.26552 29231251 PMC5826900

[9] Nalli AD, Kumar DP, Al-Shboul O, Mahavadi S, Kuemmerle JF, Grider JR, Murthy KS. Regulation of Gβγi-dependent PLC-β3 activity in smooth muscle: inhibitory phosphorylation of PLC-β3 by PKA and PKG and stimulatory phosphorylation of Gαi-GTPase-activating protein RGS2 by PKG. Cell Biochem Biophys. 2014; 70:867–80. 10.1007/s12013-014-9992-6 24777815 PMC4184950

[10] Kadamur G, Ross EM. Intrinsic Pleckstrin Homology (PH) Domain Motion in Phospholipase C-β Exposes a Gβγ Protein Binding Site. J Biol Chem. 2016; 291:11394–406. 10.1074/jbc.M116.723940 27002154 PMC4900283

[11] Palanca BJA, Avidan MS, Mashour GA. Human neural correlates of sevoflurane-induced unconsciousness. Br J Anaesth. 2017; 119:573–82. 10.1093/bja/aex244 29121298 PMC6172973

[12] Yu Y, Yang Y, Tan H, Boukhali M, Khatri A, Yu Y, Hua F, Liu L, Li M, Yang G, Dong Y, Zhang Y, Haas W, Xie Z. Tau Contributes to Sevoflurane-induced Neurocognitive Impairment in Neonatal Mice. Anesthesiology. 2020; 133:595–610. 10.1097/ALN.0000000000003452 32701572 PMC7429299

[13] Peul WC, van Houwelingen HC, van den Hout WB, Brand R, Eekhof JA, Tans JT, Thomeer RT, Koes BW, and Leiden-The Hague Spine Intervention Prognostic Study Group. Surgery versus prolonged conservative treatment for sciatica. N Engl J Med. 2007; 356:2245–56. 10.1056/NEJMoa064039 17538084

[14] Wang Z, Wang Z, Wang A, Li J, Wang J, Yuan J, Wei X, Xing F, Zhang W, Xing N. The neuroprotective mechanism of sevoflurane in rats with traumatic brain injury via FGF2. J Neuroinflammation. 2022; 19:51. 10.1186/s12974-021-02348-z 35177106 PMC8855620

[15] Day PW, Tesmer JJ, Sterne-Marr R, Freeman LC, Benovic JL, Wedegaertner PB. Characterization of the GRK2 binding site of Galphaq. J Biol Chem. 2004; 279:53643–52. 10.1074/jbc.M401438200 15471870 PMC1432089

[16] Mariggiò S, García-Hoz C, Sarnago S, De Blasi A, Mayor F Jr, Ribas C. Tyrosine phosphorylation of G-protein-coupled-receptor kinase 2 (GRK2) by c-Src modulates its interaction with Galphaq. Cell Signal. 2006; 18:2004–12. 10.1016/j.cellsig.2006.03.004 16725308

[17] Liu Z, Xia Y, Li B, Xu H, Wang C, Liu Y, Li Y, Li C, Gao N, Li L. Induction of ER stress-mediated apoptosis by ceramide via disruption of ER Ca(2+) homeostasis in human adenoid cystic carcinoma cells. Cell Biosci. 2014; 4:71. 10.1186/2045-3701-4-71 25937892 PMC4417540

[18] Min JW, Kong WL, Han S, Bsoul N, Liu WH, He XH, Sanchez RM, Peng BW. Vitexin protects against hypoxic-ischemic injury via inhibiting Ca2+/Calmodulin-dependent protein kinase II and apoptosis signaling in the neonatal mouse brain. Oncotarget. 2017; 8:25513–24. 10.18632/oncotarget.16065 28424420 PMC5421947

